# Analysis of health care claims during the peri-transfer stage of transition from pediatric to adult care among juvenile idiopathic arthritis patients

**DOI:** 10.1186/s12969-016-0107-3

**Published:** 2016-09-05

**Authors:** Melissa L. Mannion, Fenglong Xie, John Baddley, Lang Chen, Jeffrey R. Curtis, Kenneth Saag, Jie Zhang, Timothy Beukelman

**Affiliations:** 1 Department of Pediatrics, Division of Rheumatology, University of Alabama at Birmingham, 1600 7th Ave S, CPPN M10, Birmingham, AL 35209 USA; 2 Department of Medicine, Division of Clinical Immunology and Rheumatology, University of Alabama at Birmingham, 510 20th St South, FOT 802, Birmingham, AL 35294 USA; 3 Department of Medicine, Division of Infectious Disease, University of Alabama at Birmingham, 1900 University Blvd, THT 229, Birmingham, AL 35294 USA

**Keywords:** Juvenile idiopathic arthritis (JIA), Transition to adult care, Administrative claims

## Abstract

**Background:**

To investigate the utilization of health care services before and after transfer from pediatric to adult rheumatology care in clinical practice.

**Methods:**

Using US commercial claims data from January 2005 through August 2012, we identified individuals with a JIA diagnosis code from a pediatric rheumatologist followed by any diagnosis code from an adult rheumatologist. Individuals had 6 months observable time before the last pediatric visit and 6 months after the first adult visit. Medication, emergency room, physical therapy use, and diagnosis codes were compared between the pediatric and adult interval using McNemar’s test. The proportion of days covered (PDC) of TNFi for the time between last pediatric and first adult visit was calculated.

**Results:**

We identified 58 individuals with JIA who transferred from pediatric to adult rheumatology care after the age of 14. The median age at the last pediatric rheumatology visit was 18.1 years old and the median transfer interval was 195 days. 29 % of patients received NSAIDs in the adult interval compared to 43 % in the pediatric interval (*p* = 0.06). In the pediatric interval, 71 % received a JRA and 0 % received an RA physician diagnosis code compared to 28 and 45 %, respectively, in the adult interval. The median PDC for patients receiving a TNFi was 0.75 during the transfer interval.

**Conclusion:**

Individuals with JIA who transferred to adult care were more likely receive a diagnosis of RA instead of JRA and were less likely to receive NSAIDs, but had no significant immediate changes to other medication use.

## Background

Juvenile idiopathic arthritis (JIA) is a chronic disease that begins in childhood with up to 50 % of patients continuing to have active arthritis in adulthood [1–4]. JIA is an umbrella term for a heterogenous group of inflammatory arthritides; most children with JIA do not have a disease similar to rheumatoid arthritis. Accordingly, children with JIA not only have complications related to the age of onset of disease, such as growth limitations or psychosocial issues [5–7], but also have disease manifestations that are not commonly seen in rheumatoid arthritis (RA) such as silent anterior uveitis [8]. Chronic disease in childhood can affect linear growth as well as psychosocial development due to additional stress, medical experiences, and alterations in normal social and emotional development [7]. There is an emphasis on growth and development in pediatric and pediatric rheumatology training that represents an important difference between pediatric and adult rheumatologists.

As with all chronic diseases that begin in childhood, patients who are seen by a pediatric specialist in childhood must transition to an adult specialist at the age of adulthood, usually near the end of adolescence. Transition is a complicated process that requires coordination of care in a chronologic and developmentally appropriate setting in order to increase the likelihood of success [9]. Ideally the process of transition begins in early adolescence to educate and prepare individuals for the adult experiences of disease and healthcare and culminates in the transfer from a pediatric specialist to adult specialist [10]. Since the time of transition, both the age of adolescence and transfer of care, can be a high risk period for disease flares or complications, improving the transition process for young adults with special health care needs, including those with a rheumatologic diagnosis, has become a focus of multiple organizations [11]. The clinical pattern of individuals with JIA transitioning and transferring from pediatric rheumatologists to adult rheumatologists is largely unknown in the United States.

To understand the barriers that exist for patients transitioning from pediatric rheumatology to adult rheumatology, we must first know what is happening currently in clinical practice. Transition programs vary greatly across U.S. pediatric rheumatology clinics and most report that they have an informal process [12]. The goal of this study was to evaluate the health utilization patterns during the peri-transfer stage of transition. We hypothesized that individuals with JIA would receive alternate diagnoses from pediatric and adult providers (e.g. RA instead of juvenile rheumatoid arthritis (JRA)), they would have a change in medication use, more patients would receive physical therapy in the adult interval, more patients would visit an emergency room in the transfer and adult intervals, and that patients would have poor compliance with medications during the transfer period. Using national administrative claims from a large commercial US health insurer, we investigated the utilization of health care services before and after transfer.

## Methods

We performed this study using a national commercial insurance administrative claims database from January 2005 through September 2012. This claims database has been used previously to describe the trends in medication use among patients with JIA [13]. Individual providers are given unique identifiers within this database that are linked to their individual National Provider Identifier (NPI) as well as any group NPI number that the claims may be billed under. We utilized this unique identifier to identify pediatric rheumatologists and adult rheumatologists based upon their individual NPI taxonomy code. NPI taxonomy codes are assigned by the individual or institution that manages the individual NPI account [14]. Pediatric rheumatologists were identified by taxonomy code 2080P0216X (pediatric rheumatology) or 20800000X with 207RR0500X (pediatrics and rheumatology). Adult rheumatologists were identified by taxonomy code 207RR0500X without 20800000X. The providers who met the taxonomy criteria for pediatric rheumatologist were reviewed by MLM with >90 % of individuals identified by name as practicing pediatric rheumatologists by training or board certification.

**Fig. 1:**
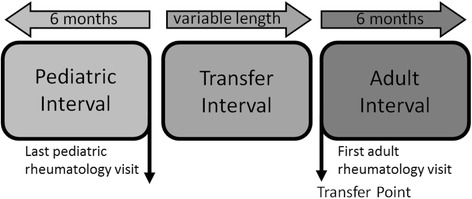
Graphical depiction of pediatric, transfer, and adult intervals

To identify the study population, we first identified individuals in the database with ≥ 2 International Classification of Diseases, 9th edition (ICD-9) codes associated with a physician encounter between 7 days and 6 months apart and consistent with JIA (714.3x (JRA), 696.0 (Psoriatic Arthritis), 720.xx (Spondyloarthritis)) or 1 diagnosis code consistent with JIA and 1 prescription for methotrexate (MTX) or a tumor necrosis factor inhibitor (TNFi). This claims definition of JIA has been used in other pharmacoepidemiologic studies [15–17]. Individuals were also required to have a JIA diagnosis code from a physician with a pediatric rheumatology NPI taxonomy code followed by a diagnosis code from a physician with a different NPI number associated with an adult rheumatology taxonomy code and without a subsequent pediatric rheumatology claim. We required individuals to have at least 6 months of continuous coverage before the last pediatric rheumatology visit (pediatric interval) and after the first adult rheumatology visit (adult interval). There was no limit on the length of the time between the pediatric and adult rheumatology visits (transfer interval). The first adult rheumatology visit was defined as the transfer point; this resulted in 3 distinct intervals, pediatric, transfer, and adult (Fig. 1). We excluded individuals who were <14 years of age at the transfer point to increase the likelihood that the transfer was due to age and not geographic relocation.

We collected demographic information including age at last pediatric visit and at transfer point, gender, US census region, calendar year of transfer point, length of transfer interval (in days), and physician diagnosis ICD-9 codes related to JIA (JRA, psoriatic arthritis, spondyloarthritis, rheumatoid arthritis) in both the pediatric and adult intervals. We determined the use of methotrexate (MTX), tumor necrosis factor inhibitors (TNFi), oral glucocorticoids, opiates, and non-steroidal anti-inflammatory drugs (NSAIDs) using pharmacy and infusion claims for each interval. Other biologics had infrequent use in this population and were not included [13]. We also identified individuals who utilized an emergency room (ER) by physician evaluation and management codes and physical therapy (PT) or occupational therapy by current procedural terminology codes. Medication, ER use, PT use, and diagnosis codes were compared between the pediatric and adult interval using McNemar’s test. The proportion of days covered (PDC) of TNFi for the transfer interval was calculated for all patients who received a TNFi during the pediatric interval. All *p* values were two sided and considered significant at values ≤ 0.05. Data analyses were performed using SAS software, Version 9.3 (copyright, SAS Institute Inc, Cary, NC, USA). The university institutional review board approved the study protocol. Although JIA is the accepted inclusive terminology for this group of diseases, JRA persists within the ICD-9 definition. Henceforth, JRA will only be used in reference to the ICD-9 code 714.3.

## Results

**Fig. 2:**
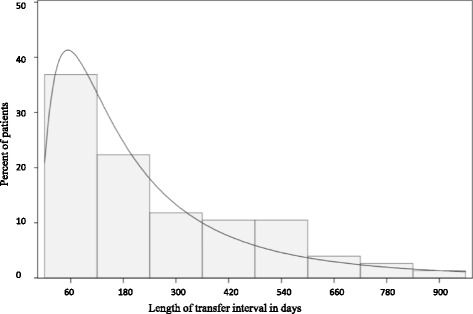
Histogram of transfer interval length in days

We identified 58 individuals with JIA who transferred from pediatric to adult rheumatology care and were at least 14 years old. A total of 2,988 individuals met the diagnosis of JIA in the available claims data; most were excluded because they did not have evidence of transfer to an adult rheumatologist. The median age at the last pediatric rheumatology visit was 18.1 years old (interquartile range (IQR) 17–19.6 years) and the median age at the first adult rheumatology visit was 18.9 years old (IQR 18–20 years). The median length of the transfer interval (time from last pediatric rheumatology visit to first adult rheumatology visit) was 195 days (IQR 89–371 days) (Fig. 2). All US census regions and years 2006–2012 were represented; 79 % of the individuals were female (Table 1).

**Table 1:** Demographics of study cohort

	Pediatric Interval	Transfer Interval	Adult Interval
Age (median (IQR))	18.1 (17–19.6)		18.9 (18–20)
Female	46 (79.31 %)		
Time, days (median (IQR)		195 (89–371)	
Census Region			
Northeast			22 (37.9 %)
South			16 (27.6 %)
Mid West			8 (13.8 %)
West			12 (20.7 %)
Calendar Year			
2005			0
2006			8 (13.79 %)
2007			12 (20.69 %)
2008			9 (15.52 %)
2009			8 (13.79 %)
2010			5 (8.62 %)
2011			13 (22.41 %)
2012			3 (5.17 %)

Individuals with JIA who transfer at >14 years old, *n* = 58. Age at last pediatric visit and first adult visit, median (IQR). Length of transfer interval in days, median (IQR); pediatric and adult intervals were 183 days by definition. U.S. Census Region and Calendar Year at first adult rheumatology visit, *n* (%)

During the 6 month pediatric interval, 33 % of patients received a TNFi, 24 % MTX (either oral or subcutaneous), 28 % oral glucocorticoids, 28 % opiates, and 43 % NSAIDs. These numbers did not significantly change compared to the 6 month adult interval where 41 % of patients received a TNFi, 21 % MTX (either oral or subcutaneous), 28 % glucocorticoids, 29 % opiates, and 29 % NSAIDs. These patients also continued to receive medications during the transfer interval with 24 % receiving a TNFi, 21 % MTX (either oral or subcutaneous), 31 % glucocorticoids, 21 % opiates, and 36 % NSAIDs (Table 2).

**Table 2:** Health Care Claims of Individuals with JIA during transfer

	Pediatric Interval	Transfer Interval	Adult Interval	*p* value
MTX PO	10 (17.24 %)	9 (15.52 %)	11 (18.97 %)	0.8
MTX SQ	4 (6.90 %)	3 (5.17 %)	1 (1.72 %)	0.1
TNFi	19 (32.76 %)	14 (24.14 %)	24 (41.38 %)	0.2
GC	16 (27.59 %)	18 (31.03 %)	16 (27.59 %)	1
OP	16 (27.59 %)	12 (20.69 %)	17 (29.31 %)	0.8
NSAID	25 (43.10 %)	21 (36.21 %)	17 (29.31 %)	0.06
ER	11 (18.97 %)	11 (18.97 %)	12 (20.69 %)	0.8
PT	14 (24.14 %)	10 (17.24 %)	11 (18.97 %)	0.4
Physician diagnosis codes				
JRA (714.3x)	41 (70.69 %)		16 (27.59 %)	<0.0001
Psoriatic arthritis (696.0)	3 (5.17 %)		3 (5.17 %)	1
Spondyloarthritis (720.xx)	16 (27.59 %)		14 (24.14 %)	0.3
RA (714.0)	0 (0 %)		26 (44.83 %)	

Number of individuals who received medications, had ≥ PT visit, ≥ 1 ER visit, and physician diagnosis codes (not mutually exclusive) during each interval, *n* (%). The pediatric interval and adult interval proportions were compared by McNemar’s test with resulting *p* value

Abbreviations: *MTX* methotrexate, *PO* oral, *SQ* subcutaneous, *TNFi* tumor necrosis factor inhibitor, *GC* glucocorticoids, *OP* opiates, *ER* Emergency Room, *PT* physical therapy, *JRA* Juvenile Rheumatoid Arthritis, *RA* rheumatoid arthritis

There was no significant difference in the number of patients receiving physical therapy or visiting an Emergency Room between the pediatric and adult intervals. The proportions of JRA and RA ICD-9 codes were significantly different between the pediatric and the adult intervals. No patient received an RA diagnosis during the pediatric rheumatology interval. Fewer patients received a JRA diagnosis (28 %) and more received an RA diagnosis (45 %) during the adult interval. There was no significant change in the number of patients who received a diagnosis for psoriatic arthritis or spondyloarthropathy between the intervals (Table 2). Of the 19 patients receiving a TNFi during the pediatric interval, the median PDC for TNFi was 0.75 (SD 0.332) during the transfer interval.

## Discussion

We were able to identify individuals with JIA who transferred from pediatric to adult rheumatology care in a national commercial health insurance population. While most patients transferred at the end of adolescence and had a median 6 month transfer interval, approximately 25 % of the children had a gap greater than 1 year between their last pediatric rheumatology visit and their first adult rheumatology visit. A clinic-based study from Canada reported that half of the individuals with JIA did not follow up with an adult rheumatologist within 2 years of their last pediatric visit [18]. A clinic based chart review of all transferred patients over 5 years from a clinic in the United Kingdom with an established transition program reported a mean age at transition of 17 years old with a median of 115 days between pediatric and adult visits [19]. The patients that we have identified are likely to have more severe disease given the high proportion of patients receiving and continuing a rheumatologic medication and may have motivated these patients to pursue rheumatology care in adulthood.

After transfer to adult care, there was very little change in the medications received by individuals with JIA. There was a non-significant trend for more patients to be on a TNFi and fewer to be prescribed NSAIDs between the pediatric and adult interval. Glucocorticoids and opiates are received in a large proportion of this population. While we do not have clinical information to know the reasons for prescription, we have published similar rates for glucocorticoid use previously [13]. In addition, there was little change from the pediatric to the transfer interval. Medication adherence can only be calculated using evidence and timing of refills in administrative claims data as it lacks any clinical information regarding patient reported adherence. In this data set, individuals who received a TNFi in the pediatric interval had a PDC of 0.75 for TNFi use during the transfer interval. A PDC of 80 % in claims data is typically interpreted as good adherence; our PDC of 75 % indicates fair adherence during the transfer interval [20].

Individuals with JIA who transferred to adult care were more likely receive a diagnosis of RA instead of JRA, but patients with psoriatic arthritis or spondyloarthropathy maintained the same diagnosis codes. JIA is classified according to the International League Against Rheumatism (ILAR) criteria [21], but ICD-9 diagnosis codes reflect an earlier classification system. Individuals with JIA continue to have JIA in adulthood; however in this US commercial claims database that is not the clinical pattern. It is unknown if the billing code reclassification is due to the limitations of ICD-9 diagnosis codes, ease of using an RA code based on available billing forms or for medication approval, or simple misclassification. This finding highlights the challenges of using claims data for longitudinal or adult outcome studies of patients with JIA that have been identified in other longitudinal studies [22]. It is unclear how transition to the ICD-10 coding system will affect this challenge to longitudinal follow up; the increase in coding options is unlikely to affect misclassification and billing habits of physicians. Most of the individuals with JIA identified continued to receive medications and did not have significant changes between intervals; patients who successfully transfer maintain disease treatment.

This study is limited by the small sample size. We did not evaluate the patients that continue to see pediatric rheumatologists into adulthood or only see adult rheumatologists from childhood as there were no differences identified in the patients who did transfer. Due to our inclusion requirements and the years for which we had data, we were limited by right censoring and the inability to discern the individuals with JIA who failed to follow up and had an unsuccessful transfer versus the patients who failed to follow up because they were in drug free remission and perhaps did not need to transfer to adult rheumatology care. The individuals who may have oligoarticular JIA and may not need adult rheumatology care cannot be uniquely identified using claims data. Those individuals who unsuccessfully transfer warrant further evaluation to determine the barriers to transition and what disease outcomes occur with unsuccessful transition, however those additional studies will have to be done via a different data source such as a longitudinal cohort study. We are unable to identify failed transitions in an administrative claims dataset due to right censoring; this would be better studied in a prospective or clinical dataset. We used data from commercial insurance that may not be generalizable to individuals with non-commercial insurance. In addition to the potential cost of health during the transition period, there are missing costs associated with the transition process and planning. The AAP recommends the use of billing for time and counseling related to transition [10]; however we are unable to assess these additional costs without clinical documentation.

## Conclusions

Understanding the current patterns of transition and transfer for individuals with JIA will contribute to the evaluation of barriers that limit patients from continuing rheumatologic care. Future studies can help identify additional risk factors or clinical features that contribute to the success or failure of transition to improve the rates of successful transition of individuals with JIA from pediatric to adult rheumatology care. There are many programs being developed to improve the process of transition and transfer from pediatric to adult rheumatology care. In this administrative claims database, the patients who were seen by an adult rheumatologist did not have significant changes to their medical management of JIA in the short-term.

## Abbreviations

ER: Emergency room
ICD-9: International classification of diseases, 9th edition
ILAR: International League Against Rheumatism
IQR: Interquartile range
JIA: Juvenile idiopathic arthritis
JRA: Juvenile rheumatoid arthritis
MTX: Methotrexate
NPI: National provider identifier
NSAIDs: Non-steroidal anti-inflammatory drugs
PDC: Proportion of days covered
PT: Physical therapy
RA: Rheumatoid arthritis
TNFi: Tumor necrosis factor inhibitor
